# Tying up loose ends: the N-degron and C-degron pathways of protein degradation

**DOI:** 10.1042/BST20191094

**Published:** 2020-07-06

**Authors:** Richard T. Timms, Itay Koren

**Affiliations:** 1 Cambridge Institute of Therapeutic Immunology and Infectious Disease, Jeffrey Cheah Biomedical Centre, Cambridge Biomedical Campus, University of Cambridge, U.K.; 2 The Mina and Everard Goodman Faculty of Life Sciences, Bar-Ilan University, Ramat Gan, Israel

**Keywords:** C-degron pathways, degron, E3 ubiquitin ligases, N-degron pathways, protein termini, ubiquitin proteasome system

## Abstract

Selective protein degradation by the ubiquitin-proteasome system (UPS) is thought to be governed primarily by the recognition of specific motifs — degrons — present in substrate proteins. The ends of proteins — the N- and C-termini – have unique properties, and an important subset of protein–protein interactions involve the recognition of free termini. The first degrons to be discovered were located at the extreme N-terminus of proteins, a finding which initiated the study of the N-degron (formerly N-end rule) pathways, but only in the last few years has it emerged that a diverse set of C-degron pathways target analogous degron motifs located at the extreme C-terminus of proteins. In this minireview we summarise the N-degron and C-degron pathways currently known to operate in human cells, focussing primarily on those that have been discovered in recent years. In each case we describe the cellular machinery responsible for terminal degron recognition, and then consider some of the functional roles of terminal degron pathways. Altogether, a broad spectrum of E3 ubiquitin ligases mediate the recognition of a diverse array of terminal degron motifs; these degradative pathways have the potential to influence a wide variety of cellular functions.

## Introduction

Protein degradation plays a critical role in essentially all major cellular processes. The ubiquitin-proteasome system (UPS) represents the major route through which the cell achieves selective protein degradation [1]. The conjugation of ubiquitin, a 76 amino acid protein, to a substrate serves as the signal for targeting to the 26S proteasome and subsequent proteolytic degradation [2,3]. Conventional ubiquitination occurs through a cascade of three enzymes: ubiquitin is activated by the E1 enzyme, transferred to an E2 ubiquitin-conjugating enzyme, and then finally conjugated to the target substrate recruited by an E3 ubiquitin ligase [4]. Overwhelmingly it is thought that specificity within the UPS is provided by the E3 ubiquitin ligases [5], of which ∼800 are encoded in the human genome.

### Degrons drive specificity within the ubiquitin-proteasome system

What are the specific molecular features of substrates that are selectively recognised by E3 ubiquitin ligases? These features — termed degrons — are defined as the minimal elements within substrate proteins sufficient to allow recognition by the degradative machinery. A key property of degrons, therefore, is their transferability: in most cases, transplantation of a degron element from an unstable protein should be sufficient to confer instability on an otherwise long-lived protein. Degrons comprise mostly short linear motifs (2–10 residues), are thought to occur preferentially in disordered regions of proteins, and can either be constitutive, promoting continuous degradation of the protein, or conditional, such as those generated by post-translational modification (PTM) or exposed following protease cleavage [6]. Despite their importance, our knowledge of degron motifs remains sparse [7], although the high-throughput genetic technologies developed to tackle this problem over the past few years hold considerable promise in accelerating degron discovery [8–12].

### Terminal degrons

Degrons can reside anywhere within a protein sequence, but the focus of this minireview are the degrons that lie at protein termini. The first degrons to be discovered — the result of remarkable work in the laboratory of Alexander Varshavsky in the 1980s — lay at the extreme N-terminus of proteins [13]. Hundreds of studies in the decades hence have delineated a network of N-degron pathways (formerly known as the N-end rule pathways [14]), through which a variety of E3 ubiquitin ligases recognise substrates via the composition of their N-termini [15]. Given the interest in these pathways, it is somewhat surprising that it took over 30 years to reveal that an analogous set of C-degron pathways operate in parallel on a suite of degron motifs located at the extreme C-termini of proteins [9,16]. Indeed, there are multiple reasons why the termini of proteins might be particularly fertile ground for degron motifs: (1) both the N- and C-termini of proteins are more likely to exist in a disordered conformation than internal regions of proteins [17], and they are also more likely to be accessible [18]; (2) free from the evolutionary constraints associated with maintaining a three-dimensional structure, the disordered termini of proteins may have greater capacity to incorporate regulatory information than do internal regions; and (3) protein termini are subject to a myriad of post-translational processing events, which can be exploited to impart conditionality to protein degradation through terminal degrons.

Here we provide a brief overview of the known N-degron and C-degron pathways, before considering some of the potential cellular functions of these degradation systems. We focus particularly on the pathways discovered in the past few years, and direct readers to excellent reviews that provide more comprehensive coverage of the Arg/N-degron [14,15] and Ac/N-degron [19] pathways. Our scope is restricted to pathways shown to occur in human cells, although N-degron pathways are also known to play important roles in plants [20–22] and bacteria [23,24].

## N-degron pathways

### Arg/N-degron pathway

**Figure 1.**
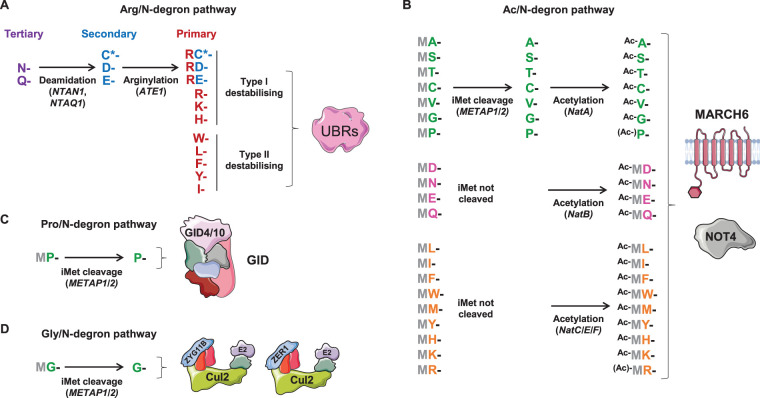
N-degron pathways. (**A**) Arg/N-degron pathway. Substrate recognition by UBR family E3 ubiquitin ligases is best understood for UBR1, which harbours two distinct substrate binding sites: one accommodates the positively charged primary type I destabilising residues (R, K and H) [71,72], whilst the second recognises the bulky hydrophobic primary type II destabilising residues (W, Y, F, L, I) [73]. Specificity for the remaining N-terminal residues comes as a result of further N-terminal processing pathways: the tertiary destabilising residues (N and Q) can be deamidated to form the secondary destabilising residues (D and E) [74,75], which are subject to N-terminal arginylation by ATE1 [76]. Oxidised cysteine (C*) is also subject to N-terminal arginylation [51]. (**B**) Ac/N-degron pathway. In certain contexts, acetylated N-termini can serve as degrons. It is estimated that up to 80% of all human proteins are N-terminally acetylated to some extent by N-acetyltransferase (Nat) enzymes, with the degree of acetylation varying depending on the sequence context [31]. (**C**) Pro/N-degron pathway. The GID E3 ligase complex targets N-terminal proline degrons. (**D**) Gly/N-degron pathway. Two Cul2 complexes target N-terminal glycine degrons via the substrate adaptors ZYG11B and ZER1.

Protein N-termini are subject to a wide variety of processing events, the most drastic of which is the cleavage of the initiator methionine (iMet) if the second residue is sufficiently small (G/A/V/C/S/T/P) [25]. Remarkably, the Arg/N-degron pathway — principally through the UBR family E3 ubiquitin ligases UBR1, UBR2 and UBR4 [26] — is capable of degrading proteins bearing aberrant N-terminal residues (the so-called primary [R/K/H/W/Y/F/L/I], secondary [D/E] and tertiary [N/Q] destabilising residues) that should not be exposed during the course of normal protein synthesis [26] (Figure 1A). Thus, the Arg/N-degron pathway likely serves as an important quality control mechanism to ensure the destruction of protein fragments [27].

The role of the Arg/N-degron pathway is not limited to protein quality control, however, and UBR family E3 ligases have now been shown to participate in a wide array of cellular processes [14]. Some notable examples include mitophagy, where the degradation of cleaved PINK1 following its retrotranslocation from mitochondria plays a critical role in sensing mitochondrial stress [28], and pyroptosis, where the UBR2- and UBR4-mediated degradation of NLRP1B is critical for inflammasome activation [29,30]. In certain cases UBR family E3 ligases can also target proteins bearing an intact initiator methionine, with these substrates enriched for proteins harbouring Arg, Lys, Leu or Ile following the initiator methionine [27]; the cellular consequences of degradation of these substrates, however, remains to be determined.

### Ac/N-degron pathway

Acetylation of the N-terminal residue is an extremely common PTM thought to occur on ∼80% of all human proteins [31]. In certain contexts the acetylated N-terminal residue can serve as a degron motif for two E3 ubiquitin ligases of the Ac/N-degron pathway: MARCH6 (also known as TEB4), a RING E3 ligase that resides in the ER membrane, and Not4, a component of the multi-subunit Ccr4-Not complex [32–34] (Figure 1B). Well-characterised substrates of this pathway include Rgs2, a regulator of G protein signalling linked to hypertension [34], and Perilipin-2, a major lipid droplet-associated protein [35]. However, it seems unlikely that the Ac/N-degron pathway plays a significant role in the global regulation of protein stability, as deletion of N-acetyltransferase enzymes has minimal effects on protein stability in yeast [10].

### Pro/N-degron pathway

The GID (glucose-induced degradation) E3 ubiquitin ligase complex was identified over 20 years ago through genetic screens in *S. cerevisiae* designed to identify genes required for the degradation of the gluconeogenic enzyme fructose-1,6-bisphosphatase (Fbp1) upon transition from growth on ethanol to glucose [36,37]. It has only recently become apparent, however, that GID is the central player in an N-degron pathway specific for proline residues (Figure 1C). Substrate recognition is achieved by the Gid4 subunit [38], which adopts a unique β-barrel structure harbouring a narrow and deep binding pocket that accommodates the N-terminal proline and the subsequent residue [39].

Intriguingly, the expression of Gid10, a Gid4 paralogue with slightly altered specificity for N-terminal proline degrons, is induced under various stress conditions [40,41]. Indeed, an elegant series of cryoelectron microscopy structures show that GID exists not as a single complex, but rather a family of E3 ligases formed from interchangeable substrate receptors that can be swapped in response to external conditions [41]. The evolutionary conservation of GID4 between yeast and man suggests that the human GID complex will also target substrates bearing N-terminal proline degrons; a priority for future studies on the Pro/N-degron pathway will be the delineation of substrates recognised by human GID4.

### Gly/N-degron pathway

We recently uncovered another N-degron pathway in which two Cullin-RING ligases (CRLs) — Cul2^ZYG11B^ and Cul2^ZER1^ — target N-terminal glycine degrons [27] (Figure 1D). CRLs are multi-subunit complexes, wherein E2 binding and substrate recruitment are carried out by distinct components [42]. The Cullin subunit itself (Cul1, Cul2, Cul3, Cul4A, Cul4B and Cul5 in human cells) serves as a central scaffold: E2 binding occurs through a RING protein (Rbx1 or Rbx2) at its C-terminus, while substrate recruitment is achieved through a substrate adaptor bound at its N-terminus. Recognition of N-terminal glycine degrons is achieved by the Cul2 substrate adaptors ZYG11B and ZER1, which are closely related (29% sequence identity). Many N-terminal glycine degrons are redundantly targeted by both adaptors, although the ZYG11B degron is shorter, comprising just N-terminal glycine and the following residue, whereas the ZER1 degron extends several residues further into the polypeptide chain and preferentially comprises amino acids with bulky side chains [27]. Two potential physiological roles for this pathway — degradation of caspase cleavage products during apoptosis and the quality control of protein *N*-myristoylation — are considered below.

## C-degron pathways

**Figure 2.**
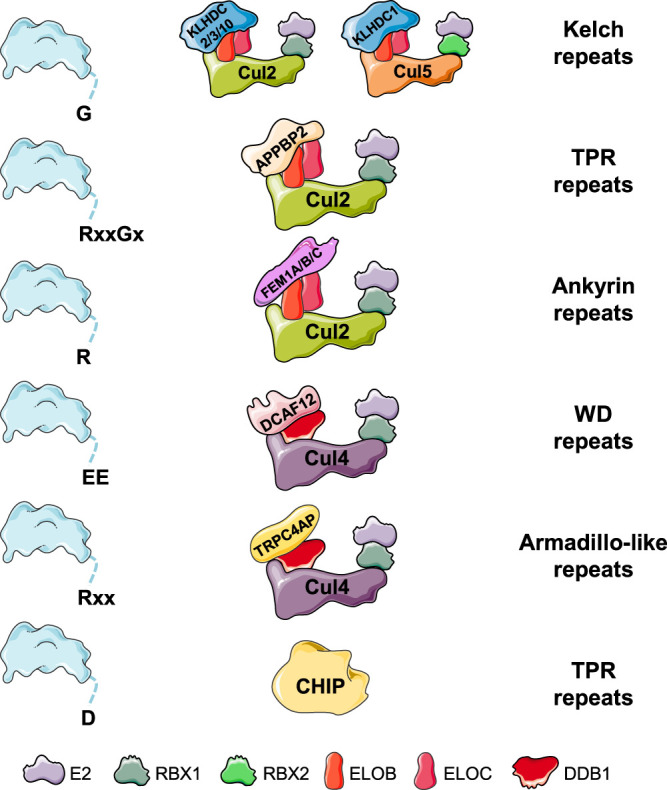
C-degron pathways. C-terminal degrons are targeted by a diverse array of E3 ubiquitin ligases, all of which employ tandem repeat domains to facilitate degron recognition. Elongin-B (ELOB) and Elongin-C (ELOC) bridge the interaction between the substrate adaptor and Cul2; DNA damage-binding protein 1 (DDB1) functions similarly in the assembly of Cul4 complexes. The full APPBP2 degron can be defined as Rx_[2–4]_Gx_[0–3]_, with RxxGx and RxxGxx serving as optimal motifs.

Here we summarise the C-degron pathways uncovered thus far (Figure 2); however, as the study of C-terminal degrons remains in its infancy, it seems likely that this list will expand in the coming years.

### Gly/C-degron pathways

A suite of CRL complexes target substrates ending with a C-terminal glycine. Best characterised are three Cul2 complexes that use related substrate adaptors of the Kelch family: Cul2^KLHDC2^, Cul2^KLHDC3^ and Cul2^KLHDC10^ [9,16]. Each adaptor appears to recognise distinct G-end motifs, with KLHDC2 showing a strong preference for −GG, KLHDC3 targeting mainly −RG and −KG, and KLHDC10 recognising −AG, −WG and −PG. In addition, KLHDC1, a Cul5 substrate adaptor, can also recognise C-terminal −GG degrons [43].

Our understanding is most advanced for KLHDC2, as crystal structures of the Kelch repeats of KLHDC2 in complex with several substrates terminating with di-glycine motifs have been solved [44]. The six-bladed β-propeller fold forms a deep central pocket which can accommodate seven amino acids from the substrate; the geometry of the pocket forces that the amino acid at the −2 position must be glycine, while only glycine and alanine can be tolerated at the −1 position. The E3 ligase-degron interaction is remarkably tight, with binding affinities in the low nanomolar range [44]. Given that KLHDC1, KLHDC3 and KLHDC10 share significant similarity with KLHDC2, it is likely that these substrate adaptors engage C-terminal glycine degrons in a very similar way.

### RxxG/C-degron pathway

Another Cul2 E3 ubiquitin ligase complex — Cul2^APPBP2^ — can also recognise C-terminal glycine degrons, but the substrate adaptor APPBP2 shows a strong preference for RxxG or RxxxG motifs (where x can be any amino acid) [9,16]. Intriguingly, whilst the Kelch-family adaptors strictly require the glycine degron to be located at the extreme C-terminus, the tetratricopeptide (TPR) repeats of APPBP2 display considerably more flexibility: the RxxG/RxxxG degron motif need only be located near (within the final ∼10 residues) to the C-terminus of the substrate, with the optimal position of the glycine residue seemingly at the −2 or the −3 position.

### Arg/C-degron pathway

Another group of related Cul2 ligase complexes — Cul2^FEM1A^, Cul2^FEM1B^ and Cul2^FEM1C^ — target substrates that terminate with arginine [9,16]. In this case, the ankyrin repeats in the FEM1-family substrate adaptors form the degron binding pocket. However, the full degron motif recognised by the different FEM1 proteins remains to be elucidated. For example, some substrates targeted by FEM1C have the arginine residue at the −2 or −3 position relative to the end [16]. The size of the degron targeted by the FEM1 proteins may also be considerably larger than for other C-degron pathways; indeed, for one example substrate transplantation of the last 25 amino acids was required to destabilise a heterologous protein [16], suggesting that internal sequences are also likely to be important for recognition. Both FEM1A and FEM1C are required for the degradation of certain substrates, suggesting that they may function as a heterodimer [9].

### C-degron pathways regulated by Cul4 complexes: R-3 and E-2 motifs

Two Cul4 E3 ubiquitin ligase complexes are also known to target C-terminal degrons: Cul4^TRPC4AP^ and Cul4^DCAF12^ [9]. Similar to APPBP2, the substrate adaptors TRPC4AP (also known as TRUSS [45]) and DCAF12 both recognise specific degron motifs near the C-terminus of the substrate. The spacing is more critical, however, with arginine required at the −3 position (R−3 motif) for recognition by the armadillo-like repeats of TRPC4AP, and a glutamic acid required at the −2 position for recognition by the WD40 repeats of DCAF12 (E−2 motif). In each case there is some degree of flexibility as to the specific residue at the C-terminal position, although a twin C-terminal glutamic acid (−EE) motif seems strongly preferred by DCAF12. An interesting class of substrates bearing this C-terminal −EE motif are multiple members of the melanoma antigen gene (MAGE) family, a set of genes which are normally expressed only in the male germline but whose aberrant expression in cancers can drive tumorigenesis [46]. Ravichandran and colleagues recently showed that the DCAF12-mediated degradation of MAGEA3 and MAGEA6 through their C-terminal −EE motifs was important for the induction of autophagy in response to nutrient deprivation [47].

### C-degron pathways not regulated by Cullins

Recognition of C-terminal degrons is not restricted to Cullin ligases. We identified example substrates wherein C-terminal alanine and valine residues could act as degron motifs in a manner that was insensitive to the Cullin-RING ligase inhibitor MLN4924 [9], showing that non-Cullin E3 ligases must also participate in C-degron pathways. Moreover, Ravalin and colleagues recently uncovered an additional C-degron pathway in which CHIP — a member of the U-box family of atypical RING E3 ligases — targets C-terminal aspartate residues generated following caspase cleavage (discussed in more detail below) [48]. Altogether, just as is the case for N-degron pathways, it is likely that (1) a broad spectrum of amino acids have the potential to act as C-terminal degrons in certain contexts, and (2) a wide array of E3 ligases from multiple families are involved.

## Cellular functions of N-degron and C-degron pathways

### Monitor protein folding, complex assembly or correct localisation

Terminal degrons could serve to monitor various aspects of protein quality control. Polypeptides harbouring terminal degrons might be able to shield these upon achieving their native fold, or following insertion into a multiprotein complex. One well-characterised example comes from the study of the COG complex, a oligomeric assembly that regulates membrane trafficking through the Golgi [49]. Cog1 is unstable when expressed in *S. cerevisiae* owing to a Ac/N-degron targeted by the ligase Not4 [33]. Remarkably, however, Cog1 is no longer unstable when co-expressed along with its binding partners Cog2 and Cog3, presumably because the N-terminal degron is now shielded from Not4 upon complex assembly. This notion of ‘conditional sequestration’ is an attractive model through which terminal degrons could play a widespread role in ensuring correct stoichiometries of protein complexes.

Similar mechanisms may ensure the correct folding of individual proteins. Indirect evidence comes from examination of ubiquitin itself, which, intriguingly, ends with a C-terminal di-glycine motif. Lin and colleagues found that this motif was not targeted by Cul2^KLHDC2^ because the C-terminal tail of folded ubiquitin is simply too short to allow it to reach into the deep KLHDC2 binding pocket; however, the addition of just two extra amino acids upstream of the -GG motif was sufficient to confer Cul2^KLHDC2^-mediated instability [16]. Thus, it seems likely that for some proteins the conditional exposure of terminal degrons to the UPS may allow the detection and removal of misfolded species.

Terminal degrons could also play a role in ensuring correct subcellular localisation of proteins. As the machinery of the UPS is not present in the secretory pathway or in mitochondria, endowing proteins destined for these sites with terminal degrons might offer an attractive strategy to ensure their correct localisation: aberrant mislocalisation to the cytosol will expose the degron to surveilling E3 ligases and result in proteasomal degradation. Indeed, proteins that localise to the secretory pathway or mitochondria are significantly more likely to harbour degrons favoured by Ubr1 at their N-terminal than are cytosolic proteins [50], and as such the Arg/N-end rule pathway may play a global role in the degradation of proteins aberrantly localised to the cytosol.

### Transduce changes in the external environment

We saw earlier how yeast exploit the Pro/N-degron pathway to regulate their metabolism in response to altered nutrient conditions by changing the subunit composition of the GID E3 ligase [41]. Further evidence of how terminal degron pathways can be used to transduce changes in the cellular environment into global changes in protein stability comes from the study of a specific branch of the Arg/N-degron pathway which operates on substrates bearing N-terminal cysteine. Cysteine was originally classified as a tertiary destabilising residue of the Arg/N-degron pathway, as its oxidation leads to its subsequent arginylation and hence UBR-mediated proteasomal degradation [51]. This reaction was considered to occur non-enzymatically until the discovery of the plant cysteine oxidases, which, under normoxic conditions, oxidise N-terminal cysteine to cysteine sulphinic acid [52,53]. This pathway mediates the adaptation to hypoxia by regulating the stability of ethylene response transcription factors [54,55]. Masson and colleagues recently showed that a similar pathway operates in human cells, identifying cysteamine (2-aminoethanethiol) dioxygenase (ADO) as a cysteine dioxygenase that is evolutionarily related to the plant cysteine oxidases [56]. Thus, ADO acts as an enzymatic oxygen sensor whose effects are transduced through altered stability of substrates bearing N-terminal cysteine by the Arg/N-end degron pathway; this mechanism has the potential to generate more rapid responses to hypoxia than the canonical transcriptional responses mediated through hypoxia inducible factor (HIF) [56].

### Regulate fate following protease cleavage

The induction of cell death during apoptosis is critically dependent on a family of proteases known as caspases, which typically cleave their substrates between an aspartic acid and a residue with a small side chain (glycine, alanine or serine) [57]. The action of N- and C-degron pathways on the resulting ‘neo-termini’ can dramatically alter the fate of the cleavage products. Ravalin and colleagues recently uncovered a C-degron pathway in which the E3 ligase CHIP targets substrates terminating with aspartic acid [48]. The C-terminal tails of the chaperones Hsp70 and Hsp90 were thought to be the primary interacting partners for the TPR repeats of CHIP [58,59], but, by assaying CHIP binding to a library of short peptides that all terminated in aspartate, the authors found that CHIP could potentially bind hundreds of C-termini exposed following caspase cleavage [48]. On the other hand, we demonstrated that hundreds of known caspase cleavage events within the cell will generate N-terminal glycine degrons targeted by Cul2^ZYG11B^ and Cul2^ZER1^ [27]. Although rarer, caspase cleavage events can also generate N-terminal residues that are substrates for the Arg/N-degron pathway, which is considered to serve a broadly anti-apoptotic role through the degradation of pro-apoptotic fragments bearing Arg/N-degrons [60].

Protease cleavage events also permit direct cross-talk between the N-degron and C-degron pathways. The deubiquitinase Usp1 is a negative regulator of the DNA damage response [61]. Exposure to genotoxic agents promotes self-inactivation of Usp1 through autocleavage [62] and proteolytic degradation [63]: the upstream cleavage product terminates with a C-terminal di-glycine motif targeted by Cul2^KLHDC2^ [16], while the downstream fragment harbours an N-terminal glutamine which serves as a tertiary destabilising residue of the Arg/N-degron pathway [64]. Thus, both N- and C-degron pathways can combine to regulate critical cellular processes following protease cleavage.

### Enforce faithful incorporation of post-translational modifications

Selenium is a critical trace element which is mostly metabolised into the amino acid selenocysteine (Sec) which is then incorporated into a subset of at least 25 proteins in human cells. Intriguingly Sec is translated by the ribosome from the codon UGA, which typically acts as a termination signal but in the correct context encodes for the incorporation of Sec into the nascent protein chain [65]. UGA/Sec decoding is not 100% efficient, however, raising the question of how the cell deals with the pool of truncated proteins resulting from failed UGA/Sec decoding. Lin and colleagues found that the amino acids encoded immediately upstream of the UGA/Sec codon could — when located at the extreme C-terminus — act as potent degrons targeted by CRL2 complexes including Cul2^KLHDC2^, Cul2^KLHDC3^ and Cul2^APPBP2^ [66]. Thus, the conditional exposure of C-terminal degron motifs ensures the faithful incorporation of Sec.

*N-*myristoylation is an important PTM in which the 14-carbon fatty acid myristate is attached to the N-terminus of a subset of eukaryotic proteins, thereby allowing them to interact with cellular membranes or to engage in protein-protein interactions [67]. Intriguingly, this modification is attached exclusively to N-terminal glycine residues, suggesting a potential role for the Gly/N-degron pathway in the surveillance of *N-*myristoylation. Upon genetic ablation or chemical inhibition of the *N-*myristoyltransferase enzymes in human cells, we found that a failure to myristoylate did indeed lead to substrate instability through the exposure of naked N-terminal glycine residues to Cul2^ZYG11B^ and Cul2^ZER1^ [27]. Notably, other common PTMs occur at defined motifs located near the termini of proteins, such as the prenylation of CAAX box motifs [68]. We speculate that additional end-directed E3 ligases may enforce the faithful incorporation of these modifications into target proteins.

All of these potential functions for terminal degron pathways — and some additional ones — are summarised in Table 1.

**Table 1.** Example cellular roles of terminal degron pathways

Function	Pathway	Substrate(s)	E3 ligase(s)	Ref(s)
Regulate complex stoichiometry	Ac/N-degron	Cog1 Hcn9	Not4 ?	[33] [33]
Regulate subcellular localisation	Arg/N-degron	Mislocalised proteins	UBRs	[50]
Transduce changes in external environment	Pro/N-degron	Gluconeogenic enzymes Fbp1, Icl1, Mdh2 and Pck1	GID	[38–41]
	fMet/N-degron	Cse4, Pgd1, and Rps22a	Psh1	[77]
	Arg/N-degron	Oxidised Cys residues	UBRs	[51–56]
Regulate stability after protease cleavage	Arg/N-degron	C-terminal fragments generated by caspase cleavage	UBRs	[62]
	Arg/N-degron	C-terminal fragments generated by calpain cleavage	UBRs	[78]
	Gly/N-degron	C-terminal fragments generated by caspase cleavage	Cul2^ZYG11B^ Cul2^ZER1^	[27]
	Asp/C-degron	N-terminal fragments generated by caspase cleavage	CHIP	[48]
	Gly/C-degron	Autocleaved Usp1	Cul2^KLHDC2^	[16]
Mediate cross-talk with autophagy	E-2/C-degron	MAGEA3 and MAGEA6	Cul4^DCAF12^	[47]
	Arg/N-degron	PINK1	UBRs	[28]
	Arg/N-degron	Various	UBRs	[79–81]
Ensure correct incorporation of PTMs	Gly/C-degron RxxG/C-degron	Selenoproteins	Cul2^KLHDC2^ Cul2^KLHDC3^ Cul2^APPBP2^	[66]
	Gly/N-degron	*N-*myristoylated proteins	Cul2^ZYG11B^ Cul2^ZER1^	[27]

## Conclusion

Collectively these findings illustrate how the UPS exploits the unique properties of protein termini to facilitate selective protein degradation. Despite intense research interest in the N-degron pathways over the past three decades, important new insights continue to be uncovered. We anticipate that the coming years will bring similar advances in our understanding of C-degron pathways, but already some common principles are emerging: for example, recognition of C-terminal degrons is achieved by tandem repeat domains — Kelch, TPR, ankyrin, armadillo and WD40 — which all have the potential to form solenoid structures [69], and terminal degron motifs are broadly under-represented across the proteome, suggesting an evolutionary pressure for proteins to avoid terminal degron pathways [9,27]. A focus of future work will be to define the cellular contexts in which these pathways operate, and to understand how dysregulation of these pathways may be relevant in disease. Controlling the stability of cleavage products may be a particularly important role, given that it is estimated that up to 70% of proteins can exist as isoforms with undocumented N- or C-termini [70]. Furthermore, just as is the case at the N-terminus, it seems likely that the action of carboxypeptidases or the deposition of PTMs will allow conditional degradation through C-degron pathways. Overall, it is clear that a more comprehensive understanding of the relationship between E3 ubiquitin ligases and their degrons would be invaluable for guiding the pharmacological manipulation of the UPS for therapeutic benefit.

## Perspective

- Regulated protein degradation by the ubiquitin-proteasome system (UPS) plays a critical role in essentially all major cellular processes. A comprehensive understanding of how the recognition of specific degrons by E3 ubiquitin ligases drives selective protein degradation will be critical if we are to attain a systems-level understanding of the UPS.
- Although our knowledge of degron motifs remains sparse, it appears that both the N- and C-termini of proteins are particularly enriched for degron motifs. A wide array of E3 ligases are now known to mediate degradation of their substrates through the selective recognition of these terminal degrons. Although in many cases the physiological roles of these pathways remain to be explored, it is clear they act on a diverse array of substrates and will impact a wide spectrum of cellular processes.
- The application of genetic approaches that allow the high-throughput identification of degron motifs and their cognate E3 ligase hold considerable promise in expanding our understanding of the degronome.

## Open Access

Open access for this article was enabled by the participation of University of Cambridge in an all-inclusive *Read & Publish* pilot with Portland Press and the Biochemical Society under a transformative agreement with JISC.

## Abbreviations

A: alanine
ADO: cysteamine (2-aminoethanethiol) dioxygenase
C: cysteine
CRL: Cullin-RING ligase
D: aspartic acid
DDB1: DNA damage-binding protein 1
E: glutamic acid
ELOB: Elongin-B
ELOC: Elongin-C
F: phenylalanine
G: glycine
GID: glucose-induced degradation
H: histidine
HIF: hypoxia inducible factor
I: isoleucine
iMet: intitator methionine
K: lysine
L: leucine
M: methionine
MAGE: melanoma antigen gene
N: asparagine
NAT: N-acetyltransferase
P: proline
PTM: post-translational modification
Q: glutamine
R: arginine
S: serine
Sec: selenocysteine
T: threonine
TPR: tetratricopeptide
UPS: ubiquitin-proteasome system
V: valine
W: tryptophan
Y: tyrosine
